# Investigation of Transient Thermal Behavior in Thyristors Under Pulse Conditions

**DOI:** 10.3390/mi16030291

**Published:** 2025-02-28

**Authors:** Guanxiang Zhang, Xiao Zhang, Junyong Lu, Yufeng Dai, Tao Ma, Bofeng Zhu

**Affiliations:** National Key Laboratory of Electromagnetic Energy, Naval University of Engineering, Wuhan 430033, China

**Keywords:** thyristor, pulse condition, temperature distribution, lateral expansion speed

## Abstract

Under pulsed discharge conditions, high-power thyristors face challenges such as an excessively high current rise rate (di/dt) and the issue of triggering front expansion, which are difficult to accurately simulate. Traditional modeling approaches often neglect the non-uniform distribution and expansion process of the internal current within the silicon wafer. In this study, we address these limitations by incorporating these critical factors into our analysis. Using a two-dimensional device–circuit co-simulation approach, we investigate the current, temperature, and thermal power distribution within the thyristor during the turn-on process under pulsed discharge conditions. Based on the simulation results, we derive the velocity equation governing the transverse expansion of the thyristor current. Furthermore, we establish a three-dimensional finite element model of the thyristor and develop a generalized extended model for complex gate structures. These models enable us to obtain the transient temperature distribution during the thyristor turn-on process under pulsed conditions. Finally, we conduct cycle surge life tests on various types of thyristors, providing valuable insights for the selection and optimization of thyristors designed for pulsed applications.

## 1. Introduction

The main semiconductor switching device used in pulse power supply systems is the thyristor [1,2,3,4]. Compared with the ordinary working condition, the pulse working condition has the characteristics of high voltage, large current, and high power, so we must pay attention to the heating problem of thyristors. In addition to voltage breakdown, the di/dt and surge current damage of thyristors are related to their transient thermal characteristics [5]. In some studies, it has been found that for every 10 °C increase in thyristor junction temperature, its failure rate almost doubles [6]. Therefore, it is necessary to study the transient thermal characteristics of thyristors.

After passing through the pulse current, the junction temperature in the thyristor will rise rapidly, and there is a heat concentration point on the silicon wafer. Local overheating on the silicon wafer may lead to abnormal operation or even failure of the device, which seriously threatens the reliability of the system. According to the reliability research report on the power electronic system, the failure rate of power equipment in the power conversion system is the highest, accounting for about 31% [7]. Through the anatomy analysis of the damaged devices, it is found that more than 70% of the damage is caused by excessive di/dt. The main reason for high di/dt causing damage to thyristors is thermal fatigue damage [8]. Under pulse discharge conditions, the di/dt of thyristors can reach several hundred amperes per microsecond, which can easily cause an excessive temperature rise in local areas during the turn-on process, leading to device damage. Therefore, studying the transient thermal characteristics of thyristors and obtaining the local peak temperature in the devices during the turn-on process is crucial for ensuring the safe and reliable operation of the entire transmission system.

At present, the measurement methods of thyristor junction temperature are mainly divided into two types: one is to use the temperature sensor to directly measure the junction temperature in the device under test; the second is to use the thermal parameters to indirectly obtain the junction temperature change in the thyristor [9,10]. At present, the on-state voltage drop thermal method is more mature. In addition to the experimental measurement, the junction temperature in the thyristor can also be obtained by establishing the relevant model. Traditional modeling methods mainly include the finite element method [11,12,13,14] and thermal impedance model method [15,16,17,18]. Researchers [19] have studied the transient thermal characteristics distribution in thyristors but have simplified the cross–concentric-circular gate to a simple concentric circle, making it difficult to obtain the true local peak temperature in the conduction area in the early stage of thyristor triggering, and the simulation results have difficulty being equivalent to the actual operating conditions. To study the thermal behavior of thyristors under high blocking voltage and repeated discharge frequency, researchers established a thermal calculation model including an electric heating model and a finite element model. However, they did not consider the lateral dynamic expansion process of thyristors after turn-on during the cyclic charge–discharge process [20]. In the conventional modeling approach, the non-uniform distribution of current inside the wafer is ignored and the current is treated as flowing uniformly throughout the wafer. In addition, it is assumed that the heat production is also uniform throughout the silicon wafer. However, in the pulse discharge condition, there is a trigger front extension problem due to the excessive current rise rate di/dt [21]. For high-power thyristors, after the gate is triggered, the thyristor will start to conduct from the cathode near the gate and gradually expand to the whole thyristor. At this stage, the temperature of the silicon wafer will rise rapidly, resulting in local peak temperature.

This paper considers the front extension of the trigger and the nonuniform distribution of the current inside the silicon wafer. Device–circuit co-simulation is performed by establishing a physical model related to the thyristor conduction mechanism, a two-dimensional device model, and an equivalent circuit model of pulse discharge. The distribution of electric field, current, and temperature during the turn-on process of the thyristor under pulse condition is analyzed, and the expansion velocity equation of the thyristor is extracted. Then, considering the heat transfer in the silicon wafer, molybdenum wafer, and copper wafer, three-dimensional finite element thyristor model and gate opening expansion model are established. Combined with the expansion velocity equation of the thyristor, the temperature distribution in the thyristor in the turn-on process is simulated and analyzed. The research in this paper can provide guidance for the selection and application of thyristors in pulse operating conditions, as well as the optimization design of gate patterns.

## 2. Two-Dimensional Non-Uniform Model of Thyristors

### 2.1. Establishment of the Model

**Figure 1:**
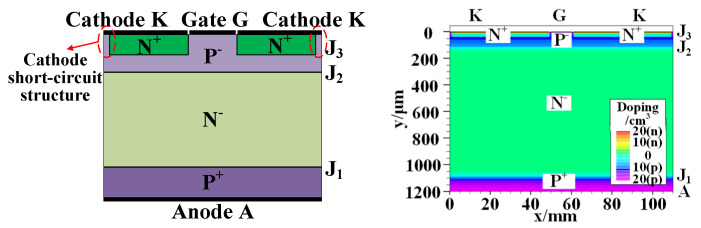
Internal structural cross-section (**left**) and full-scale two-dimensional device model of thyristor (**right**).

A thyristor is a four-layer three-terminal semiconductor device, which consists of three PN junctions: the anode junction (J_1_), gate junction (J_2_) and cathode junction (J_3_). Based on the conduction mechanism of the thyristor and its internal doping, a full-size two-dimensional device model of the thyristor is built. Figure 1 is a cross-section of the thyristor chip. The basic structural parameters of the device and the doping distribution in each region can be seen from the figure. The specific parameters are shown in Table 1.

Parameters such as the width of the N^−^ base region and doping concentration [22], are designed through some theoretical formulas and empirical parameters, as shown in Table 2. In particular, the following parameters are calculated based on (100) orientation silicon wafers.

The simulation of thyristor devices is mainly to obtain the electrothermal characteristics of the corresponding devices by solving a series of mathematical and physical partial differential equations, and the accuracy of the simulation is closely related to the physical model chosen [23,24,25]. The physical model established in the subsequent simulation of this article is shown in Table 3.

The meaning of the variables and parameters involved in the above physical model is shown in Table 4. The parameters not listed in the table are all real constants.

During the turn-on process of the thyristor, electrons and holes will drift under the action of the electric field. As the electric field increases, the drift velocity does not always increase and tends to a certain maximum value. Therefore, it is necessary to select the mobility model to describe the drift velocity of the carriers.

There is a carrier recombination process in the turn-on process of the thyristor, so the relevant recombination model needs to be selected. The recombination model mainly includes SRH recombination, radiation recombination, and Auger recombination. SRH plays a leading role in indirect bandgap semiconductors such as Si, and radiation recombination plays a leading role in direct bandgap semiconductors such as GaAs, while Auger recombination plays a leading role in high-doping and high-injection conditions because it requires high carrier concentration [24]. Considering that the thyristor model established in this paper is made on the Si substrate and there will be a large carrier injection in the cathode and anode, it is necessary to select the relevant SRH recombination and Auger recombination models.

In addition to the mobility model and the recombination model, the impact ionization model must be selected because avalanche breakdown occurs at the reversely biased J_2_ junction. In order to obtain the transient temperature distribution characteristics on the whole silicon wafer, it is necessary to select the lattice self-heating and energy balance model.

**Figure 2:**
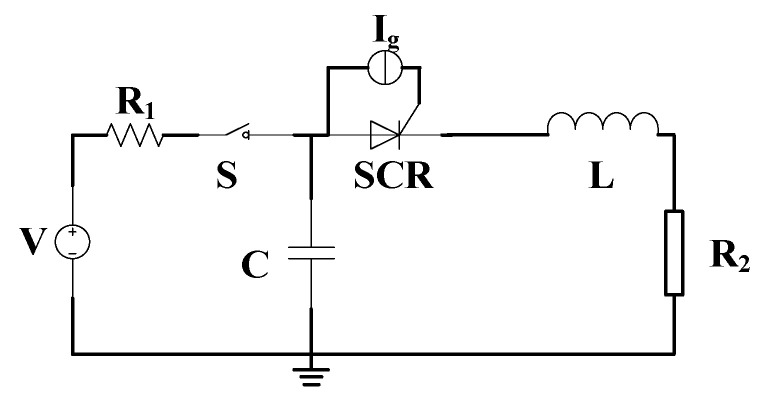
Equivalent circuit model of pulse discharge.

In order to simulate the pulse condition, the equivalent circuit model of pulse discharge is established, as shown in Figure 2. In the figure, **V** is the charging device, **R**_1_ is the line resistance, **S** is the high-voltage switch, **C** is the energy storage capacitor, ***SCR*** is the thyristor, **I**_g_ is the gate trigger current, **L** is the equivalent inductance, and **R**_2_ is the equivalent load.

The simulation process is as follows: First, the model parameters of the thyristor (including doping concentration, base region widths, etc.) are determined based on theoretical formulas and empirical data. Next, the device is divided into a mesh, with finer grid spacing in critical areas (such as near the P–N junction) to improve simulation accuracy. Then, the physical models required for simulating the electrothermal characteristics of the thyristor are established as the foundation for solving. Simultaneously, a pulse discharge circuit model is constructed, connecting the device’s electrodes (anode, cathode, gate) to circuit nodes to achieve bidirectional electrothermal coupling. Finally, an external trigger signal is applied to the gate to observe the current spreading process and temperature distribution changes.

### 2.2. Solution and Analysis of the Model

**Figure 3:**
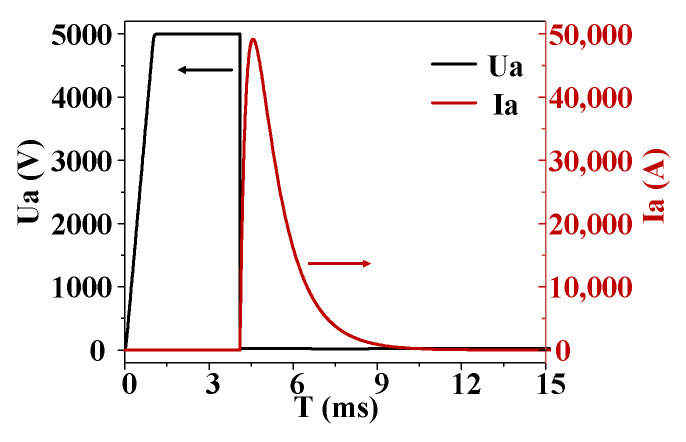
Voltage and current curves of thyristor under pulse discharge.

Through the joint simulation of the device–circuit, the voltage and current of the thyristor under pulse discharge are obtained, as shown in Figure 3. It can be seen that when the trigger current is applied to the gate at 4.1 ms, the voltage of the thyristor decreases rapidly and approaches a minimum value after about 2 μs. The current rises relatively slowly, reaching its maximum value of 49 kA after about 460 μs. The curve of the dissipation power is consistent with the change trend of current, reaching its maximum value of 822 kW after about 413 μs.

**Figure 4:**
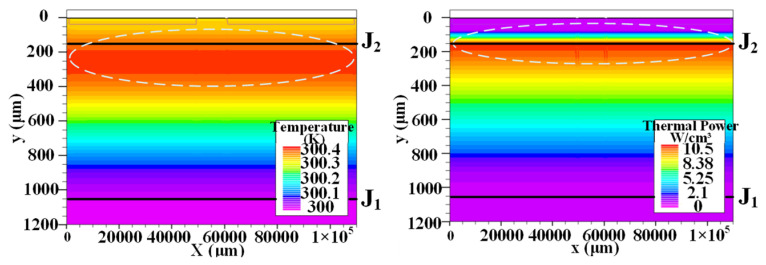
The temperature (**left**) and heat power (**right**) distribution in thyristor without triggering.

When the trigger current is not applied to the thyristor, the temperature and thermal power distribution in the thyristor are shown in Figure 4. At this time, only a small leakage current flows through the thyristor, and the thermal power consumption is very small. The maximum thermal power is only 10.5 W/cm^3^, the temperature change is less than 1 K, and the maximum temperature is located in the region below the J_2_ junction.

**Figure 5:**
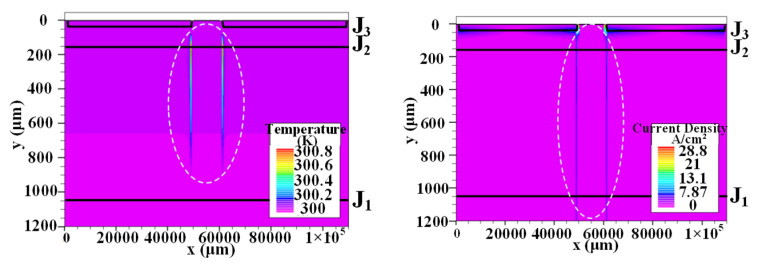
The distribution of temperature (**left**) and current density (**right**) in thyristor at 0.7 μs.

The temperature and current density distribution at *t* = 0.7 μs after applying the trigger current to the gate are shown in Figure 5. In the local region of the cathode near the gate side, the minority carrier injection current in the J_3_ junction increases, and the electrons will be extracted to the N^−^ region under the action of a high electric field and cross the N^−^ region, causing the reinjection of holes, as shown in Figure 5 (right). Due to the small initial conduction area, the heat dissipation increases rapidly, and its maximum power density reaches 782 kW/cm^3^. However, the trigger current is applied to the gate for a short time, so the thyristor has not yet begun to conduct, and the temperature rise is not obvious.

**Figure 6:**
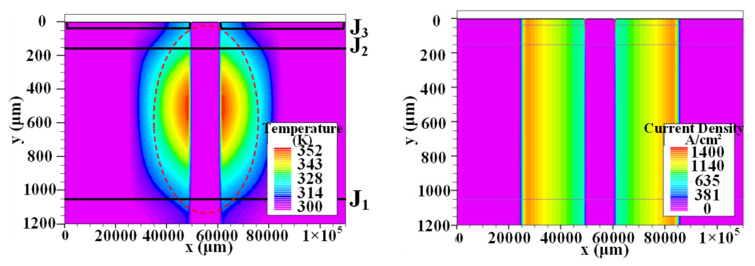
The distribution of temperature (**left**) and current density (**right**) in thyristor at 346 μs.

At *t* = 346 μs, the temperature and current density distribution in the thyristor are shown in Figure 6. When the local conduction current is large enough, the conduction area gradually expands. Comparing Figure 5 (right) and Figure 6 (right), the conduction area of the thyristor increases significantly. As can be seen in Figure 6, the maximum temperature reaches 352 K, with the peak temperature point located in the middle of the initial conduction region. Therefore, under pulse discharge conditions, the size of the initial conduction area of the thyristor is extremely important, and when the initial conduction area is too small, thermal breakdown is likely to occur in this area.

### 2.3. Extraction of Spread Velocity Equation

**Figure 7:**
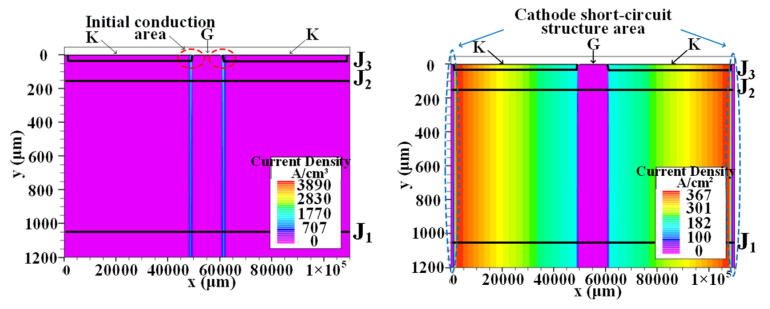
Current density distribution in thyristor during lateral expansion at 9 μs (**left**) and 1469 μs (**right**).

After applying the trigger current to the gate of the thyristor, the current density distribution in the thyristor at different times is shown in Figure 7. After the thyristor triggers conduction, the thyristor is not initially in the full region conduction state. When the trigger current flows through the thyristor base, only part of the cathode area near the gate side is turned on, and the rest is still in the blocking state, where only a small leakage current flows through. After partial conduction of the thyristor, a transverse electric field occurs because the unopened region of the J_2_ junction is still reverse-biased. Moreover, the current in the local conduction region rises rapidly, and a large number of electrons and holes are gathered near the conduction region of the J_2_ junction. These carriers will expand laterally to the unopened region through diffusion. At the same time, the existence of the transverse electric field will also make the holes in the P^−^ base region flow to the unopened region through drift motion. The movement of these carriers will form a transverse current, just as a trigger current is applied to the unopened region. When the current rises above the holding current, the unopened region will change from blocking to conduction. Through the transverse expansion process of the carriers in the conduction region, the conduction region is gradually extended to the entire element. At this time, the current through the cathode short-circuit structure region can be ignored, as shown in Figure 7.

The lateral current expansion process in the thyristor is influenced by a variety of factors, including the forward blocking voltage, base carrier lifetime, base width, conduction current density, current rise rate, and gate pattern [30,31,32,33]. It will have an important impact on the di/dt, short-term surge current, and turn-on loss of the device. However, as an important parameter of the expansion process, there is currently no unified analytical formula for the lateral expansion speed. At present, some scholars measure the expansion speed by the probe method and the infrared method, both of which require etching a small observation hole on the cathode contact. This is difficult to achieve for high-power thyristors that are packaged and applied to several kilovolts of high voltage and tens of thousands of amperes of pulse large current.

This study aims to obtain the expansion speed of the thyristor through simulation. In both two-dimensional and three-dimensional thyristor models, parameters such as forward blocking voltage, temperature, and base region width are identical. However, the two-dimensional model does not consider the impact of gate shape on the lateral current expansion process, which needs to be further considered in three-dimensional modeling. Under high electric fields, the lateral biased electric field effect is considered the primary influencing factor, and the lateral current expansion speed is mainly related to the current density in the conductive part of the thyristor. The expansion speed increases with the rise in local current density. Therefore, based on the simulation results of the current lateral expansion process of the full-size thyristor model shown in Figure 7 and the expansion speed lateral bias model, the equation of expansion speed versus current density was fitted and obtained by extracting a large amount of simulation data for verification:

*v* = 36.4 In(*J*) − 550, (1)

where *J* is the average current density of the turned-on region in A/m^2^, and *v* is the expansion speed of the thyristor in m/s.

## 3. Establishment and Solution of Three-Dimensional Non-Uniform Model of Thyristor

### 3.1. Establishment of Three-Dimensional Finite Element Model

**Figure 8:**
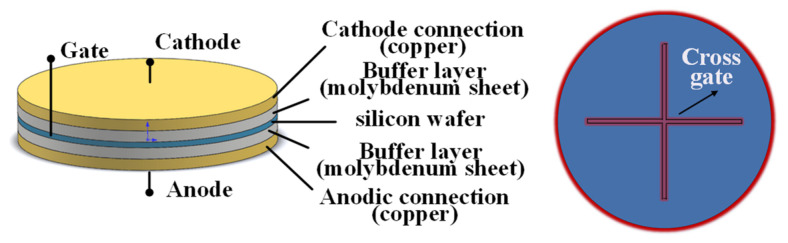
Three-dimensional structure model of thyristor (**left**) and cross-shaped gate pattern of silicon wafer (**right**).

Figure 8 shows the three-dimensional structure model of the thyristor. In the thyristor, the silicon wafer is sandwiched; at the bottom is the copper wafer connected to the anode, and at the top is the copper wafer connected to the cathode, with a buffer layer (molybdenum wafer) added between the copper and silicon wafer. In order to ensure the full turn-on of the thyristor, it is important to improve the di/dt tolerance of the thyristor or to optimize the design of the gate pattern of the silicon.

### 3.2. Establishment of Cross-Shaped Gate Model

**Figure 9:**
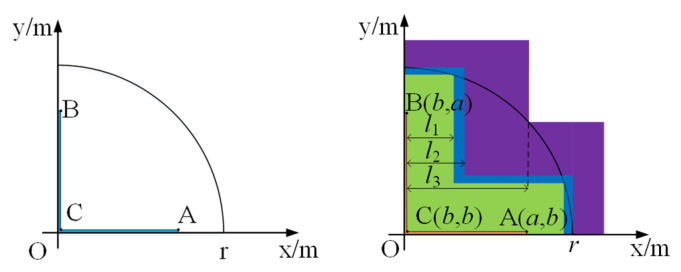
Coordinate position of quarter wafer model (**left**) and schematic diagram of the critical point in gate expansion stage (**right**).

Assuming the expansion process, the cross-shaped gate pattern remains unchanged. The X–Y coordinate system is established with the cross center as the coordinate origin. Since the silicon wafer as well as the gate pattern are axially symmetrical, the expansion process of the gate in all four quadrants is the same, and it is simplified to a quarter silicon wafer model, as shown in Figure 9.

The corresponding point *A* coordinates are (*a*,*b*), point *B* is (*b*,*a*), point *C* is (*b*,*b*), and *r* is the radius of the silicon wafer, where *a* and *b* satisfy Equations (2) and (3), respectively.

(2) $$a=a_{1}+l(t_{i}),$$

(3) $$b=b_{1}+l(t_{i}),$$

where *l*(*t*_i_) is the total spread distance at time *t*_i_, *a*_1_ and *b*_1_ are the initial positions of the gate, and their units are m.

As shown in Figure 9, when point *A* and point *B* extend to the first boundary of the thyristor at the same time (i.e., the boundary of the green area), point *A* satisfies Equation (4):

(4) $$a^{2}+b^{2}=r^{2},$$

where *l*(*t*_i_) is the total spread distance at time *t*_i_, *a*_1_ and *b*_1_ are the initial positions of the gate, and their units are m.

Substituting Equations (2) and (3) into Equation (4), the corresponding total expansion distance *l*_1_ can be obtained:

(5) $$l_{1}=0.5\left(\sqrt{2r^{2}-{\left(a_{1}-b_{1}\right)}^{2}}-a_{1}-b_{1}\right),$$

As the expansion continues, when it extends to the second boundary (i.e., the blue region boundary), the total expansion distance *l*_2_ is (*r* − *a*_1_).

When the conduction region continues to expand to the third boundary (i.e., the purple region boundary), the lateral expansion process of the thyristor ends and the whole area of the silicon wafer is turned on. At this time, the coordinates of point *C* are (*b*,*b*), satisfying the relationship shown in Equation (6):

(6) $$b^{2}+b^{2}=r^{2},$$

Substituting Equation (3) into Equation (6), the total extended distance *l*_3_ can be obtained:

(7) $$l_{3}=\sqrt{2}r/2-b_{1},$$

Through the three boundary positions defined above, the thyristor gate expansion process can be divided into four parts. With the increase in the total expansion distance *l*(*t*_i_), the corresponding area *s*(*t*_i_) on the silicon wafer can be expressed as the following function:

(1) When 0 ≤ *l*(*t*_i_) < *l*_1_, the following obtains:

(8) $$s(t_{i})=4b(2a_{1}+l(t_{i})-b_{1})-4b_{1}(2a_{1}-b_{1})\;,$$

(2) When *l*_1_ ≤ *l*(*t*_i_) < *l*_3_, the following obtains:

(9) $$\begin{array}{cc} s(t_{i})= & 4b(2a_{1}+l(t_{i})-b_{1})-8(ab-0.5b\sqrt{\left(r^{2}-b^{2}\right)}\\ & -0.5r^{2}(0.5\pi -\arccos\left(b/r\right)-\arccos\left(a/r\right)),\\ & -0.5a\sqrt{\left(r^{2}-a^{2}\right)})-4b_{1}(2a_{1}-b_{1}) \end{array}$$

(3) When *l*_3_ ≤ *l*(*t*_i_), the following obtains:

(10) $$s(t_{i})=\pi r^{2}$$

### 3.3. Solution and Analysis of the Model

In this paper, the gate length *a*_1_ is 40 mm, the width *b*_1_ is 1 mm, the radius of the thyristor silicon wafer, molybdenum wafer, and copper wafer is 110 mm, and the thickness is 1 mm, 3 mm, and 3 mm, respectively. The ambient temperature *T_a_* was set to 22 °C.

**Figure 10:**
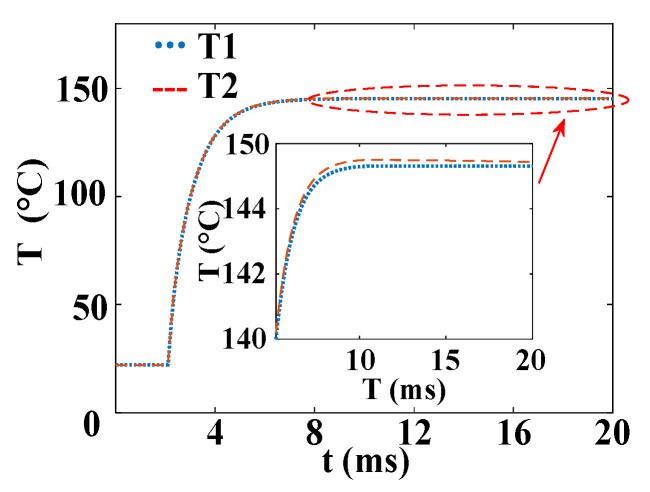
Comparison of temperature curve between formula calculation and finite element simulation.

In order to eliminate the numerical calculation error caused by the grid and time step, as well as to verify the correctness of the simulation of temperature distribution on the silicon wafer, a single-silicon-wafer three-dimensional thyristor model is established. The boundary conditions of thermal insulation are set on the surface of the silicon wafer. It is assumed that the dissipated power is converted into heat, which is compared with the results calculated by the endothermic theory, as shown in Figure 10. As can be seen in the figure, T1 is the transient peak temperature profile of the thyristor during the turn-on process obtained by theoretical calculation, and T2 is the transient peak temperature profile obtained by finite element simulation. The error between the two is very small. The maximum temperature obtained from the theoretical derivation is 145.3 °C, and the maximum temperature obtained from the finite element simulation is 145.5 °C. The error is only 0.2 °C, which confirms the accuracy of the finite element simulation results.

**Figure 11:**
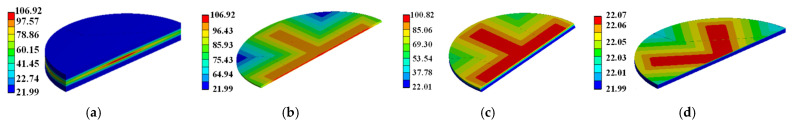
Temperature distribution. (**a**) The whole model at 5.3 ms. (**b**–**d**) Silicon/molybdenum/copper wafer at 5.3 ms.

In order to simulate the heat transfer from silicon to molybdenum and copper wafer, considering the heat dissipation of molybdenum and copper wafer, the temperature distribution during the turn-on process under pulse discharge is simulated based on the three-dimensional model of the thyristor shown in Figure 8. The room temperature was set to 22 °C. The temperature distribution on the thyristor at *t* = 5.3 ms after applying the trigger current is shown in Figure 11.

From Figure 11a, it can be observed that at *t* = 5.3 ms, the heat is mainly concentrated on the silicon wafer, with a local maximum temperature of 106 °C. Only a small part of the heat is transferred to the molybdenum wafer. There is almost no heat on the copper wafer, and its temperature is basically the same as the room temperature. This is mainly because both silicon and molybdenum have relatively small thermal conductivity, so very little heat is transferred from the silicon wafer. The temperature distributions for the silicon, molybdenum, and copper wafers are presented in Figure 11b–d.

**Figure 12:**
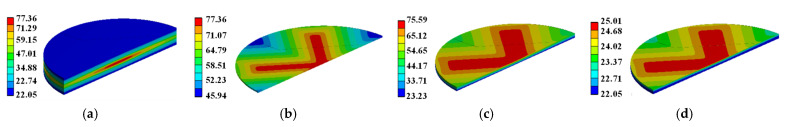
Temperature distribution. (**a**) The whole model at 20 ms. (**b**–**d**) Silicon/molybdenum/copper wafer at 20 ms.

At *t* = 20 ms, a large amount of heat has been transferred from the silicon wafer to the molybdenum wafer, while the molybdenum wafer conducts a little heat to the copper wafer, as shown in Figure 12a. Where the temperature distribution on the silicon, molybdenum, and copper wafers is shown in Figure 12b–d, the maximum temperature on the silicon wafers decreased to 77 °C and the maximum temperature on the copper wafers increased to 25 °C.

**Figure 13:**
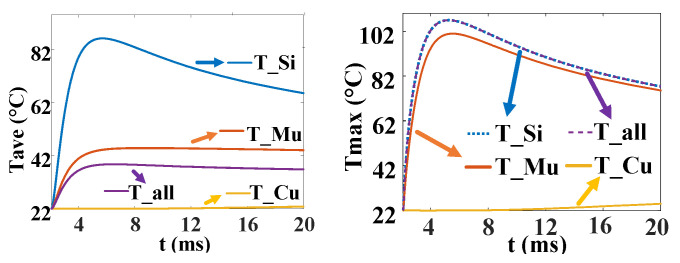
Average (**left**) and maximum (**right**) temperature curve of each region in thyristor turn-on process.

From Figure 13, the initial conduction area is small, and the temperature rises fast. After that, the temperature rises more and more slowly because of the increase in the conduction area. During the discharge process, the maximum average temperature of the silicon is 86 °C, and the time is *t* = 5.5 ms. At this time, the overall average temperature of the device is about 38 °C, the average temperature of the molybdenum is about 44 °C, and the temperature of the copper is 22 °C, basically unchanged. Therefore, during the whole pulse discharge process, the heat in the thyristor is mainly concentrated on the silicon wafer, and there is a local maximum temperature of 106 °C, which is located in the initial conduction region near the gate.

### 3.4. Establishment of Complex Gate Model

**Figure 14:**
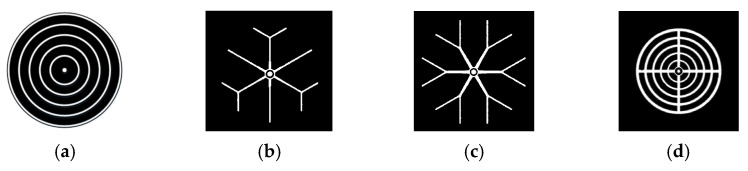
Design of different gate patterns for thyristors. (**a**) Concentric circle. (**b**) Snowflake. (**c**) Branch. (**d**) Cross–concentric circle.

The cross-shaped gate previously created and the concentric-circle gate shown in Figure 14a, because of their simple and regular shapes, can be modeled to obtain an analytical expression for the thyristor conduction area at each moment of the expansion process. However, the three complex gate patterns shown in Figure 14b–d are usually unable to obtain specific analytical expressions in the expansion process.

At present, most of the expansion modeling of complex gates is simplified to some relatively simple gate patterns. The simplification of the gate pattern will bring an error in the calculation of the instantaneous local conduction area. When the error is used to calculate the instantaneous expansion speed, it will bring more error. With the accumulation of errors, the results will have large errors. Therefore, it is necessary to establish a set of modeling methods for complex gate patterns.

**Figure 15:**
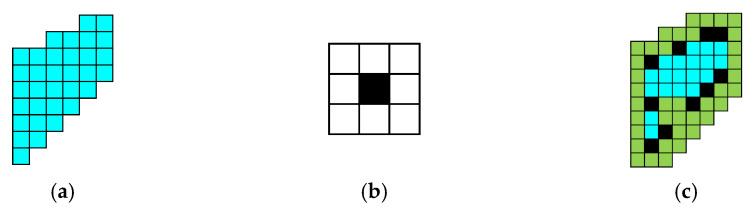
Expansion operation diagram. (**a**) Original image. (**b**) Template. (**c**) Inflated image.

Consulting the data, it is found that the thyristor gate expansion process has some similarities with the corrosion expansion algorithm in image processing [34,35]. The algorithm is commonly used in image processing, mainly for black and white binary image operation. The image erosion operation is similar to “domain reduction”, while the expansion operation is similar to “domain expansion”. The main consideration here is the expansion operation, and the schematic diagram of the expansion operation is shown in Figure 15. Figure 15a–c are the original image (a), template (b), and the inflated image (c).

**Figure 16:**
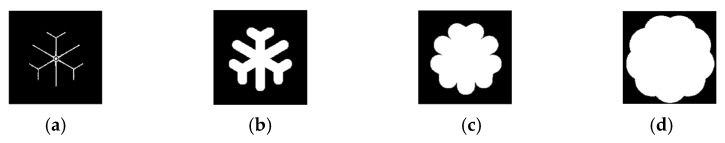
Expansion diagram of snowflake gate. (**a**) time t_1_. (**b**) time t_2_. (**c**) time t_3_. (**d**) time t_4_. (t_1_ < t_2_ < t_3_ < t_4_).

In this paper, the corrosion and expansion operations in digital image processing are applied for the first time to the simulation of the thyristor gate turn-on and expansion process. Based on the algorithm, a generalized model is established for the complex gate pattern of the thyristor, which can be linked with the finite element three-dimensional model to simulate the turn-on and expansion process of all the complex gate patterns of the thyristor, so as to obtain the temperature evolution distribution in the thyristor in the turn-on process under the pulsed condition. Figure 16 shows the application of the generalized expansion model established in this paper for snowflake gate patterns, which shows the expansion of the gate at different moments.

### 3.5. Solution and Analysis of the Model

**Figure 17:**
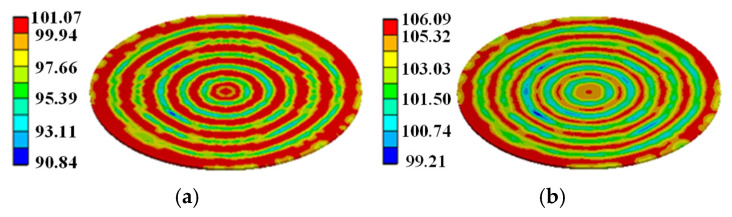
Temperature distribution on simplified concentric-circle gate silicon wafer: (**a**) at 5.3 ms, (**b**) at 15 ms.

**Figure 18:**
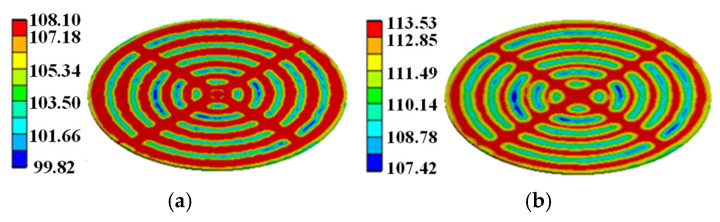
Temperature distribution on –concentric-circle gate silicon wafer: (**a**) at 5.3 ms, (**b**) at 15 ms.

In order to demonstrate that the simplification of the gate pattern of the pulsed thyristor has a large effect on the temperature simulation under the consideration of the turn-on expansion process of the thyristor, based on the same pulse condition, the transient temperature distributions in the cross–concentric-circle gate shown in Figure 14d and the simplified concentric-circle gate shown in Figure 14a are simulated, and the results are shown in Figure 17 and Figure 18. The local maximum temperature in the cross–concentric-circle-type gate is larger than that in the simplified concentric-circle-type gate.

**Figure 19:**
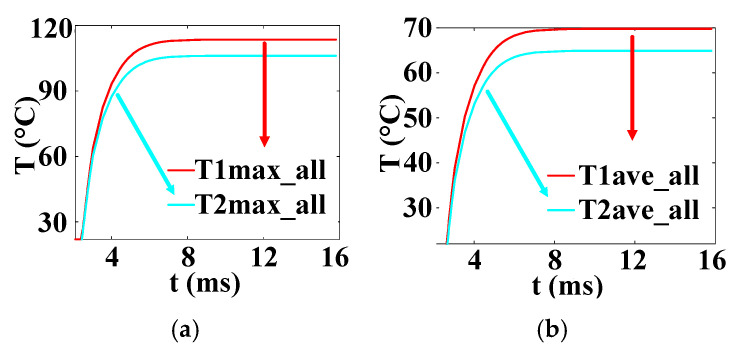
Temperature curves (T1 is the cross–concentric-circle-type gate and T2 is the concentric-circle-type gate). (**a**) Maximum temperature curve. (**b**) Average temperature curve.

**Figure 20:**
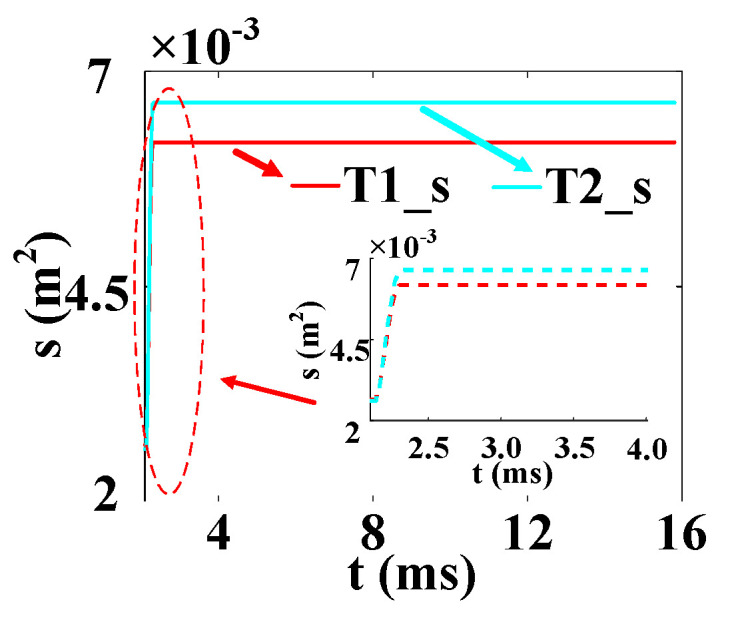
Local conduction area curve of cross–concentric-circle gate and simplified concentric-circle gate (T1 is the cross–concentric-circle-type gate and T2 is the concentric-circle-type gate).

The temperature variation curves of the cross–concentric-circle-type gate and the simplified concentric-circle-type gate thyristor are shown in Figure 19. The instantaneous local conduction area variation curve is shown in Figure 20, where T1 is the cross–concentric-circle-type gate and T2 is the concentric-circle-type gate. From Figure 19, it can be seen that the local maximum temperature in the cross-type gate electrode thyristor is 106 °C, and the maximum average temperature is 65 °C, while the local maximum temperature in the cross–concentric-circle-type gate electrode thyristor is 113 °C, and the maximum average temperature is 70 °C. Simplifying the cross–concentric-circle-type gate electrode to concentric-circle-type gate electrode gives rise to an error in the simulation results. The difference in the average temperature is 5 °C and the difference in the local maximum temperature is 7 °C, which will have an effect on the selection and design of the thyristor. As can be seen in Figure 20, the expansion speeds of the two types of thyristors are nearly identical. However, due to the larger gate area of the cross–concentric-circle gate, the current-carrying area of the cathode is reduced, resulting in a higher temperature rise in the cross–concentric-circular structure. This is also the primary source of error when simplifying the cross–concentric-circular structure to a concentric-circular structure for temperature field simulation.

**Figure 21:**
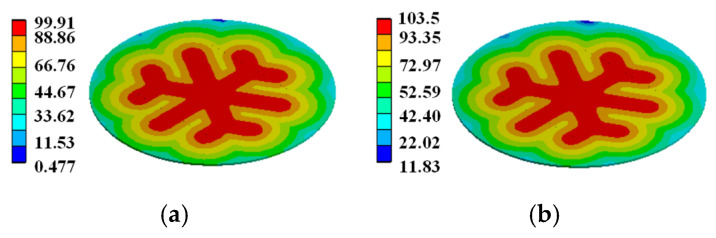
Temperature distribution on snowflake gate silicon wafer: (**a**) at 5.3 ms, (**b**) at 15 ms.

**Figure 22:**
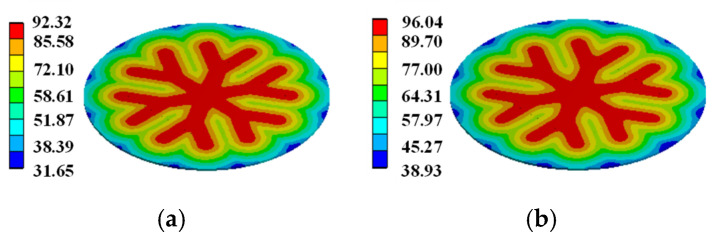
Temperature distribution on branch gate silicon wafer: (**a**) at 5.3 ms, (**b**) at 15 ms.

For the complex gate pattern shown in Figure 14, the transient temperature distribution in the thyristor during the turn-on process of the same pulse condition is simulated. The simulation results are shown in Figure 18, Figure 21 and Figure 22. It can be seen from the diagram that with the increase in time, the heat on the thyristor expands to the cathode region, and there is a local maximum temperature in the initial opening region.

**Figure 23:**
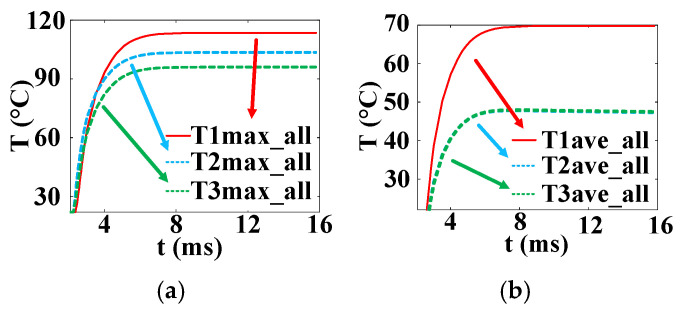
Temperature curves of three kinds of complex gate (T1 is the cross–concentric-circle gate, T2 is the snowflake gate, and T3 is the branch gate). (**a**) Maximum temperature curve. (**b**) Average temperature curve.

Figure 23 is the temperature change curve of the five-layer structure of the three complex gate thyristors. T1 is the temperature curve of the cross–concentric-circle gate, T2 is the temperature curve of the snowflake gate, and T3 is the temperature curve of the branch gate. It can be found that the average temperature in the cross–concentric-circular gate is as high as 70 °C under the same pulse condition, and the average temperature in the snowflake gate and the branch gate is almost equal, at about 48 °C. However, the local maximum temperatures in the snowflake type and the tree branch type are different, which are 103 °C and 96 °C, respectively. The local maximum temperature in the cross–concentric-circular gate is still the highest, reaching 113 °C. In summary, the thermal characteristics of the branch-gate electrode during the thyristor turn-on process are the best, followed by the snowflake-gate electrode, and the thermal characteristics of the cross–concentric-circular-gate electrode are the worst.

**Figure 24:**
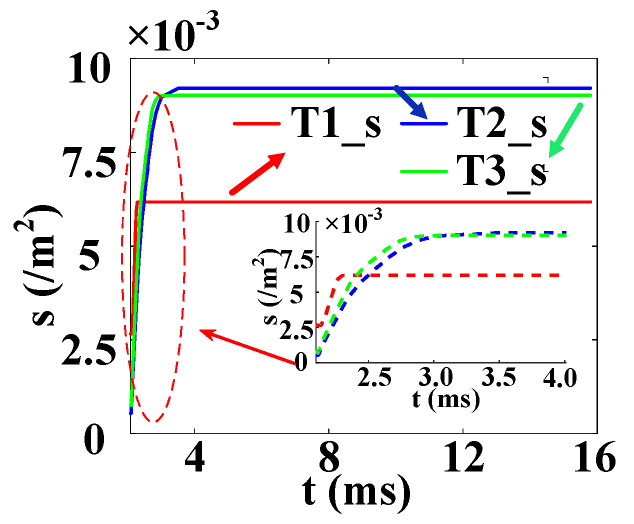
Local conduction area curves of three kinds of complex gate (T1 is the cross–concentric-circle gate, T2 is the snowflake gate, and T3 is the branch gate).

In the following, combined with the change curve of the local conduction area, the reasons for the temperature change in the three complex gates are analyzed in depth. In Figure 24, T1, T2, and T3 are the change curves of the instantaneous local conduction area in the cross–concentric-circle-, snowflake-, and branch-gate thyristors, respectively. It can be seen from the figure that although the cross–concentric-circle-type gate expands faster than the snowflake-type gate and the branch-type gate, the temperature rise will increase instead because the gate area is too large, resulting in a decrease in the cathode flow area. It can be seen from Figure 19 that for snowflake and branch gates, the instantaneous conduction area of the branch gate has been slightly larger than that of the snowflake gate. This is to say that the expansion speed of the branch-type gate is faster than that of the snowflake-type gate, so the local maximum temperature is relatively low. However, the total cathode area of the branch gate is slightly smaller than that of the snowflake gate, so the temperature rise in the branch gate is higher after both devices are fully turned on. Therefore, with the increase in time, the average temperature in the snowflake gate is lower than that in the branch gate. Therefore, for the thyristor, it is not that the larger the gate area is, the lower the temperature rise is. Due to the decrease in the cathode area, the temperature rise in the thyristor with a large gate area may be higher.

Compared with the traditional model, the thyristor model established in this paper takes into account the problems of the trigger front expansion and the nonuniform distribution of the current inside the silicon wafer, and at the same time establishes an extended model of the complex gate structure, which is able to reflect more realistically the temperature distribution during the turn-on process of the thyristor under pulse discharge. Experimental verification of the established thyristor model is considered below.

In pulsed-power discharge experiments, there are two main difficulties for thyristor junction temperature measurement: one is that the currently produced pulsed thyristors are packaged and cannot be measured by direct contact in high-voltage pulsed-cycling high-current conditions, and the other is that the time to reach the peak temperature is in milliseconds, which requires that the response time of the temperature sensor be at the μs level. Existing mature infrared thermal imaging, platinum resistance, thermocouples, and other temperature measurement methods are difficult to be applied in the pulsed high-current conditions of the packaged thyristor components. Therefore, it is considered to build a pulse discharge circuit to evaluate the performance of different-gate thyristor devices produced directly by high-voltage pulse-cycling conditions.

**Figure 25:**
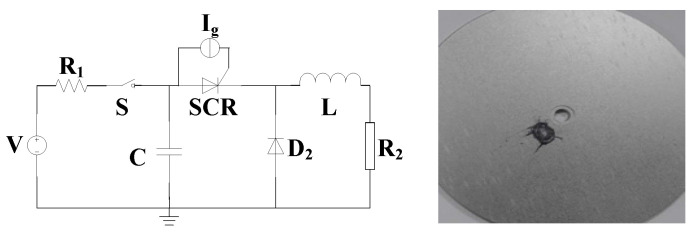
Pulse discharge circuit diagram (**left**) and failure diagram of cross–concentric-circular thyristor (**right**).

Finally, the pulse discharge circuit shown in Figure 25 was established to measure the leakage current in different-gate-type thyristors under a cyclic surge high current of 90 kA, where D2 is a stack of silicon diodes. In the surge experiment, dozens of times later, the cross–concentric-circular thyristor experienced breakdown damage, with the damage location close to the center point and an obvious melting phenomenon. After analysis, the actual cause of device damage is the accumulation of heat in the center of the device, which leads to an increase in local temperature and a decrease in the ability to withstand surge currents, resulting in high-current breakdown failure.

**Figure 26:**
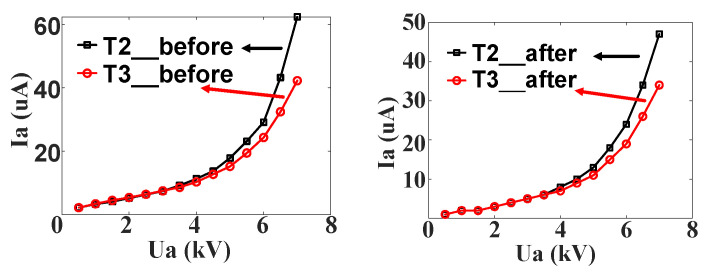
Leakage current curve before and after life test.

Under the same test conditions, the snowflake-type and branch-type thyristors did not experience breakdown failure in the surge test. Their leakage current results before and after 5000 times of surge life test are shown in Figure 26, respectively, where T2 is the test result of the snowflake thyristor and T3 is the test result of the branch thyristor. From the figure, it can be found that after the life test, the leakage current curves of the snowflake and branch type are almost the same before and after the test, and there is no degradation and failure. As in the simulation results, these two types of thyristors are indeed more superior in terms of surge current tolerance compared to the cross–concentric-circle type and can be effectively used in 90 kA surge current environments. The cross–concentric-circle type, snowflake type, and branch type, three kinds of gate thyristors that underwent an equivalent-cycle surge life test, proved the above simulation in the conclusion of the truth: as the gate area of thyristors of the same size at surge conditions becomes larger, the final temperature rise in the device may not necessarily decrease lower due to the decrease in cathode area. This requires that the leakage current and turn-on speed of the thyristor must be considered comprehensively, and the turn-on expansion process of the gate cannot be ignored in the simulation of the temperature characteristics of the thyristor and device design. At the same time, it indirectly proves the accuracy of the thyristor simulation model established in this paper, which can be used to guide the selection and optimization design of the device.

## 4. Conclusions

In this paper, a two-dimensional device–circuit model of thyristor is established, and the turn-on process of the thyristor under pulse discharge is well simulated. It is found that the highest temperature distribution in the thyristor is located in the lower region of the J_2_ junction at the initial stage after trigger. However, the temperature rise is not obvious, because it is only the leakage current at this time, and the thermal power consumption is very small. When the thyristor is first turned on near the gate area, due to the small initial conduction area, the thermal power consumption increases rapidly, the temperature rises rapidly, and the thermal breakdown failure is prone to occur in the initial opening area. At the same time, the transverse expansion velocity equation of the thyristor current is extracted. The velocity is mainly related to the forward blocking voltage of the thyristor, the carrier life of the base region, the width of the base region, temperature, and other factors, and has nothing to do with the gate pattern.

(1) In order to clarify the influence of different gate patterns on the thermal distribution of thyristors, a numerical model of expansion is established for simple and regular gate patterns such as cross patterns, and accurate numerical solutions can be obtained. Aiming at the irregular complex gate pattern, a general expansion model is established, which can accurately simulate the turn-on and expansion process of the complex gate pattern;
(2) Considering the nonuniform distribution of current and temperature inside the silicon wafer, a three-dimensional finite element model of the thyristor is established. The transient temperature distribution in thyristors with cross–concentric-circle-, snowflake-, and branch-gate patterns during the turn-on process under pulse conditions is simulated. The results show that the branch-type thyristor has the lowest local and average temperature rise and the best thermal characteristics. The average temperature rise in the snowflake type is approximately equal to that in the branch type, and the maximum temperature rise is slightly higher than that in the branch type. The local and average temperature rise in the cross–concentric-circle type is the highest, and the thermal characteristics are the worst;
(3) The cyclic surge current test was carried out on different types of thyristors. The cross–concentric-circle type evidenced thermal breakdown failure after dozens of times, while the leakage current test of the branch type and snowflake type was still normal after 5000 surge discharge cycles. Combined with the cyclic surge test and simulation results, the impact of the gate area on the temperature rise in the thyristor is further analyzed. Due to the decrease in the cathode area, the thyristor with a large gate area may have a higher temperature rise.

Compared with the traditional model, the model established in this paper considers the expansion of the trigger front and the nonuniform distribution of the internal current in the silicon wafer. It can more truly reflect the temperature distribution during the turn-on process of the thyristor under pulse discharge and has guiding significance for the selection and optimization design of the thyristor applied in the pulse condition.

## Figures and Tables

**Table 1:** Specific parameters of 2D device model.

Region	Length (μm)	Width (μm)	Doping Type	Max Doping (cm^−3^)
Anode (P^+^, bottom)	110,000	150	P	10^20^
Drift region (N^−^, base)	110,000	900	N	10^13^
Base region (P^−^, base)	110,000	112	P	10^17^
Cathode (N^+^, top)	98,000	38	N	10^20^

**Table 2:** Modeling formulae of thyristor model.

Parameter	Value	Physical Meaning	Unit
a	0.75	Constant	
c	100	Constant	
n	4~6	Constant	
A	5.29 × 10^−5^	Constant	
q	1.60 × 10^−19^	Electronic charge	C
*L_p_*	${\left(D_{p}\tau_{p}\right)}^{1/2}$	The diffusion length of holes	cm
*V_B_*	$c\rho_{n}^{a}$	Avalanche breakdown voltage of PN junction	V
*V_BF_*	$V_{B}{\left(1-\alpha_{1}\right)}^{1/n}$	Forward turning voltage	V
*ρ_n_*	${\left(2anc^{-0.5n}L_{p}A^{-1}V_{BF}^{0.5(n-1)}\right)}^{2{\left(an+1\right)}^{-1}}$	The resistivity of the base region	Ω·cm
*X_m(N1)_*	$A\;{\left(\rho_{n}V_{BF}\right)}^{0.5}$	Widening of the space charge region in the N^−^ base region	cm
*W_n(N1)_*	$X_{m(N1)}\left(1+{(\mathrm{an})}^{-1}\right)$	The width of the N^−^ base region	cm
*N_n_*	${\left(\rho_{n}\mu_{n}q\right)}^{-1}$	Doping concentration in the N^−^ base region	cm^−3^

**Table 3:** Physical model of thyristor [23,24,25,26,27,28,29].

Parameter	Value
Klaassen low-field mobility model	$\mu_{n0}^{-1}=\mu_{nL}^{-1}+\mu_{nD}^{-1}+\mu_{nA}^{-1}+\mu_{np}^{-1}$ $\mu_{p0}^{-1}=\mu_{pL}^{-1}+\mu_{pD}^{-1}+\mu_{pA}^{-1}+\mu_{pn}^{-1}$
Saturation velocity model	$\mu_{n}\left(E\right)=\mu_{n0}{\left[1+{\left(\mu_{n0}EV_{SATN}^{-1}\right)}^{\beta_{n}}\right]}^{-{\beta_{n}}^{-1}}$ $\mu_{p}\left(E\right)=\mu_{p0}{\left[1+{\left(\mu_{p0}EV_{SATp}^{-1}\right)}^{\beta_{p}}\right]}^{-{\beta_{p}}^{-1}}$
Klaassen composite model	$\tau_{n}^{-1}=(\tau_{0.n}^{-1}+C_{SRH.n}N){\left(300T_{L}^{-1}\right)}^{\delta_{n}}+\left(C_{Aug.n}p^{2}\right){\left(T_{L}\;/\;300\right)}^{\xi_{n}}$ $\tau_{p}^{-1}=(\tau_{0.p}^{-1}+C_{SRH.p}N){\left(300T_{L}^{-1}\right)}^{\delta_{p}}+\left(C_{Aug.p}n^{2}\right){\left(T_{L}\;/\;300\right)}^{\xi_{p}}$
Bandgap narrowing model	${\Delta V}_{g0}=V_{1}\left(ln\left(NN_{0}^{-1}\right)+\sqrt{\left(ln{\left(NN_{0}^{-1}\right)}^{2}+C\right)}\right)$
Collision ionization model	$\alpha_{n}\left(E\right)=A_{n}e^{\left(-B_{n}E^{-1}\right)}$ $\alpha_{p}\left(E\right)=A_{p}e^{\left(-B_{p}E^{-1}\right)}$
Lattice self-heating model	$C\frac{\partial T_{L}}{\partial t}=\nabla \left(\lambda \nabla T_{L}\right)+H$

**Table 4:** Physical model parameters and related symbols.

Symbol	Physical Meaning	Unit
*μ_n0_* (*μ_p0_*)	The total mobility of low field electrons (holes)	cm^2^/V·s
*μ_nL_* (*μ_pL_*)	Electron (hole) mobility caused by lattice scattering	cm^2^/V·s
*μ_nD_* (*μ_pD_*)	Electron (hole) mobility caused by donor scattering	cm^2^/V·s
*μ_nA_* (*μ_pA_*)	Electron (hole) mobility caused by host scattering	cm^2^/V·s
*μ_np_* (*μ_pn_*)	The effect of hole (electron) scattering on electron (hole) mobility	cm^2^/V·s
*E*	Local electric field	V/cm
*V_SATN_* (*V_SATP_*)	The saturation velocity of electrons (holes)	cm/s
*τ_n_* (*τ_p_*)	Concentration-dependent electron (hole) lifetime	s
*τ_0.n_* (*τ_0.p_*)	The intrinsic lifetime of electrons (holes)	s
*n* (*p*)	Concentration of electrons (holes)	cm^−3^
*C_SRH.n_* (*C_SRH.p_*)	Electron (hole) SRH recombination coefficient	cm^3^/s
*C_Aug.n_* (*C_Aug.p_*)	Auger recombination coefficient of electrons (holes)	cm^6^/s
*T_L_*	Lattice temperature	K
*N*	Total doping concentration	cm^−3^
Δ*V_g0_*	Bandgap width	mV
*α_n_(E)* (*α_p_(E)*)	Electron (hole) ionization rate	cm^−1^
*C*	Heat capacity per unit volume	J/K
*λ*	Thermal conductivity	W/(m·K)
*H*	Heat generation rate	W/m^3^

## Data Availability

The original contributions presented in this study are included in the article. Further inquiries can be directed to the corresponding author.
